# Remnants of an Ancient *Deltaretrovirus* in the Genomes of Horseshoe Bats (Rhinolophidae)

**DOI:** 10.3390/v10040185

**Published:** 2018-04-10

**Authors:** Tomáš Hron, Helena Farkašová, Robert J. Gifford, Petr Benda, Pavel Hulva, Tamás Görföl, Jan Pačes, Daniel Elleder

**Affiliations:** 1 Institute of Molecular Genetics, The Czech Academy of Sciences, Videnska 1083, 14220 Prague, Czech Republic; helena.fabryova@gmail.com (H.F.); hpaces@img.cas.cz (J.P.); daniel.elleder@img.cas.cz (D.E.); 2 MRC-University of Glasgow, Centre for Virus Research, 464 Bearsden Road, Glasgow G12 8TA, UK; Robert.Gifford@glasgow.ac.uk; 3 Department of Zoology, Charles University, Vinicna 7, 12844 Prague, Czech Republic; petr_benda@nm.cz (P.B.); pavel.hulva@natur.cuni.cz (P.H.); 4 Department of Zoology, National Museum (Natural History), Vaclavske nam. 68, 11579 Prague, Czech Republic; 5 Department of Biology and Ecology, University of Ostrava, Chitussiho 10, 71000 Ostrava, Czech Republic; 6 Department of Zoology, Hungarian Natural History Musem, Baross Utca 13, 1088 Budapest, Hungary; gorfol.tamas@nhmus.hu

**Keywords:** retrovirus, evolution, *Deltaretrovirus*, endogenous retrovirus, genomics, bats

## Abstract

Endogenous retrovirus (ERV) sequences provide a rich source of information about the long-term interactions between retroviruses and their hosts. However, most ERVs are derived from a subset of retrovirus groups, while ERVs derived from certain other groups remain extremely rare. In particular, only a single ERV sequence has been identified that shows evidence of being related to an ancient *Deltaretrovirus*, despite the large number of vertebrate genome sequences now available. In this report, we identify a second example of an ERV sequence putatively derived from a past deltaretroviral infection, in the genomes of several species of horseshoe bats (Rhinolophidae). This sequence represents a fragment of viral genome derived from a single integration. The time of the integration was estimated to be 11–19 million years ago. This finding, together with the previously identified endogenous *Deltaretrovirus* in long-fingered bats (Miniopteridae), suggest a close association of bats with ancient deltaretroviruses.

## 1. Introduction

Retroviruses (family Retroviridae) have an unusual replication strategy in which a copy of the viral genome is integrated into the genome of the infected host cell. This integrated copy (referred to as a “provirus”) is then expressed by the host cell machinery to generate infectious retrovirus particles. When retroviral infection occurs in a germline cell (i.e., sperm, eggs or early embryo), integrated proviruses can potentially enter the germline of the host species, so that they are vertically inherited from one generation to the next as host alleles called endogenous retroviruses (ERVs). These sequences are common in the genome of most vertebrates (especially mammals) and have exerted an important impact on the evolution of host genomes [1].

ERVs provide useful information about the long-term evolutionary relationships between retroviruses and their vertebrate hosts. Indeed, it is really only via the retrospective evidence of ERVs that we can be certain retroviruses have been infecting humans and other mammalian species for many millions of years.

Of the seven retroviral genera, only two contain viruses that are known to cause disease in humans—the genus *Lentivirus* contains the human immunodeficiency viruses (HIV-1 and HIV-2), while the genus *Deltaretrovirus* contains the human T-lymphotropic viruses (HTLVs). ERVs derived from either of these genera are extremely rare. But while ERVs derived from lentiviruses are only uncommon, ERVs derived from deltaretroviruses are vanishingly rare [2].

In a recent paper, we described the first unambiguous example of an endogenous *Deltaretrovirus* [3]. We identified Miniopterus Endogenous Retrovirus (MINERVa) in the genome of long-fingered bats (Chiroptera: Miniopteridae). In this study, we uncover further evidence of ancient deltaretroviruses infecting ancestral bat species, through the identification of a remnant deltaretrovirus ERV in the genome of horseshoe bats (Rhinolophidae).

## 2. Materials and Methods

### 2.1. Analysis of Sequencing Data

Basic Local Alignment Search Tool [4] was used to query sequence datasets from the Sequence Read Archive (SRA) and whole genome sequences database (WGS) available at the National Center for Biotechnology Information (NCBI). Specifically, genomic SRA data (SRR2278680) and transcriptomic SRA data (SRR2273931, SRR2273875, SRR2273816, SRR2273762, SRR2273740, SRR2273739, SRR2273738) from *R. sinicus* and WGS data from *R. sinicus* (GCA_001888835) and *R. ferrumequinum* (GCA_000465495) were used.

### 2.2. Genomic DNA Samples

The bat tissue samples (parts of the wing membrane and/or pectoral muscle) were obtained from museum specimens deposited in collections of the National Museum and Charles University (both in Prague, Czech Republic) and the Hungarian Natural History Museum (Budapest, Hungary). The bat species were identified with respect to their external and cranial morphological traits. Total DNA from the specimens was isolated using phenol-chloroform extraction method.

### 2.3. PCR and Sequencing

To assess the presence of ChirDelta2 sequence in various bat species, one internal amplicon (primer sequences: 5′-GGGCTCAGAAGCGAATGTCCT and 5′-CGACAGGCAGGCAGAGAACTT) and two amplicons covering 5′ and/or 3′ virus-genome junctions (primer sequences: 5′-GAAATTCATAGCATTGCAGGCCTAG and 5′-AGGCCTGTGTCTGTCAGGTGGT; 5′-TGCTGTTCTTTTTCCAGATCCCTTA and 5′-GACACTCTACCACCGGCCTGAC) were used. In samples from *R. sinicus* and *R. acuminatus*., two overlapping fragments covering whole ChirDelta2 sequence were amplified (primer sequences: 5′-GACCCAAAAATCTATGGGATGCC and 5′-GCAGGATATGACGGCTGAAGGT; 5′-TGCTGTTCTTTTTCCAGATCCCTTA and 5′-TTGATTTCCCGAAGCTGTTCGT), isolated from agarose gels and directly sequenced. The PCR amplifications were performed with a 1:200 mixture of Deep Vent and Taq polymerases (both from NEB, Ipswich, MA, USA) in all cases.

### 2.4. Provirus Copy-Number Analysis

ddPCR system QX200 (Bio-Rad, Hercules, CA, USA) was used to accurately quantify the ChirDelta2 proviral copies in rhinolophid samples. The reactions containing 10 ng DNA were treated for droplet generation and PCR-amplified according to the manufacturer’s manual. The amplified samples were analyzed by droplet reader and QuantaSoft program (Bio-Rad, Hercules, CA, USA) with thresholds set manually. Absolute copy-number values of ChirDelta2 locus were normalized to the values obtained for reference locus in rhinolophid genome. Primers used for ChirDelta2 locus: 5′-GGGCTCAGAAGCGAATGTCCT and 5′-CGACAGGCAGGCAGAGAACTT. Primers used for reference locus: 5′-TGGCCAACTTGTTGCTGAAC and 5′-AAGTCTCTGACCCTGCAGTTC.

## 3. Results

Previously, we showed that a simple homology-based search is a powerful tool for identifying novel retroviral lineages endogenized in vertebrate genomes [3]. Using this approach, we detected a sequence in the genome of *R. sinicus* that disclosed similarity to MINERVa—a recently described endogenous *Deltaretrovirus* from the genome of *Miniopterus* bats [3]. This sequence represents the only significant match to MINERVa in all available vertebrate whole genome sequences (WGS) and nucleotide sequence databases deposited in the National Center for Biotechnology Information (NCBI), with a BLAST e-value of 2 × 10^−20^.

**Figure 1:**
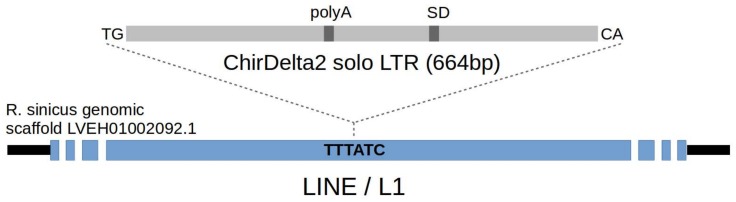
Schematic of the endogenous deltaretroviral sequence integrated in genome of rhinolophid bats. Canonical TG and CA dinucleotides at the ends of LTR sequence, polyadenylation site (polyA) and splice donor site (SD) are depicted. Integration of LTR sequence into the LINE element (blue) present in rhinolophid genome is indicated by dashed lines. 6-bp long target site sequence is marked by bold letters—this sequence has been duplicated upon the integration of provirus.

This putative deltaretroviral sequence is localized on a single scaffold (GenBank accession no. LVEH01002092; Figure S1) of the *R. sinicus* genome assembly (GenBank accession no. LVEH00000000). Sequence comparison with the MINERVa provirus showed that it represents a single viral long terminal repeat (LTR) sequence flanked by target-site duplications (TSD) that are six base pairs (bp) in length—this being consistent with other deltaretroviruses [5]. Repeatmasker analysis [6] revealed that the surrounding part of the scaffold is represented by a LINE/L1 element, into which the proviral sequence has been integrated (Figure 1). Moreover, almost the entire 7134 bp-long scaffold is comprised of L1 fragments, which precludes further description of the insertion locus in a chromosomal context. Based on the close homology to the MINERVa *Deltaretrovirus*, we named the virus this sequence is derived from ‘chiropteran *Deltaretrovirus* 2′ (ChirDelta2), while the locus itself is refered to as ERV-ChirDelta2.1. When referring to the insertion in a particular species, we append the species name (or an abbreviation of it): e.g., ERV-ChirDelta2.1-*R. sinicus*.

ERV-ChirDelta2.1 consists of only a single LTR sequence, and therefore does not contain any internal coding sequence possessing retroviral genes. Such structures are frequently generated via recombination between the 5′ and 3′ LTRs of an integrated provirus [7]. Since LTR sequences are highly variable even within retroviral genera [8], phylogenetic analysis of the evolutionary relationships between ChirDelta2 and other deltaretroviruses is not feasible. Nevertheless, two lines of evidence point to the deltaretroviral origin of ChirDelta2 sequence: firstly, the homology with previously described MINERVa LTR extending across majority of the sequence (sequence identity 53.6%; Figure S2); and secondly, a markedly elevated cytosine content which is a hallmark of deltaretroviral sequences (Figure S3) [9].

A common approach to dating ERV insertions involves measuring divergence between the 5′ and 3′ LTR sequences, which are identical at the time of insertion [10]. However, this approach cannot be applied here, since we have only identified a solo LTR. As an alternative to estimating—at least approximately—the time since ChirDelta2 integration, we evaluated the distribution of ChirDelta2 sequence across related bat species.

Whole genome sequence assemblies are available for two species of rhinolophid bats—*R. sinicus* and *R. ferrumequinum*. We did not detect any sequences related to ChirDelta2 in the *R. ferrumequinum* assembly. We also examined the raw Illumina sequencing data of *R. sinicus* and *R. ferrumequinum* and obtained the same results. The apparent absence of ChirDelta2 in the *R. ferrumequinum* genome suggested that integration occurred after diversification of rhinolophid species. To investigate the presence of ChirDelta2 integration more thoroughly we performed PCR-based screening of a panel of genomic DNAs isolated from *Rhinolophus* and other closely related species. For screening, we used one set of primers designed to the internal region of ChirDelta2 and two sets of primers targeting 5′ and 3′ provirus-host junctions, respectively. We analyzed 19 rhinolophid species and two species of the Rhinopomatidae family, which belong to the same clade as Rhinolophidae, superfamily Rhinolophoidea (Table S1). Fourteen rhinolophids were positive for ChirDelta2 sequence, whereas both rhinopomatids and 5 rhinolophids were negative. Using primers spanning the provirus-host junctions, we also determined that all positively tested rhinolophid species harbor the ChirDelta2 sequences at identical, orthologous, positions. We could not identify an empty ChirDelta2 integration site in the negatively scoring bat species, because the integration is located within repetitive L1 element, precluding PCR specificity.

To confirm the sequence obtained from database we also PCR-amplified and sequenced ChirDelta2 from two positive rhinolophids—*R. sinicus* and *R. acuminatus*. Comparing these two sequences with the insertion in *R. sinicus* genome assembly, we detected only a few randomly distributed single-nucleotide mismatches. These sequences were deposited in the GenBank database (accession no. MG983744, MG983745). Analysis of these sequences revealed strong splice donor site localized in the 3′ region of ChirDelta2 (Figure 1 and Figure S1), which is a typical feature of deltaretroviral LTRs [11]. This motif contains a point mutation in the *R. sinicus* GenBank sequence.

**Figure 2:**
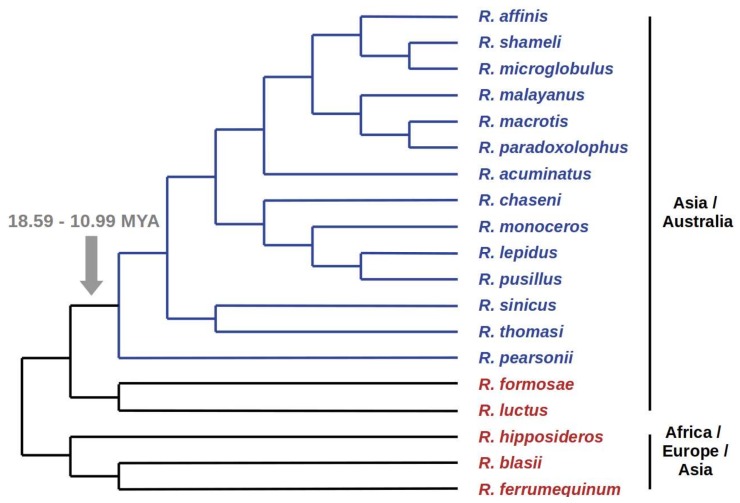
The presence of ChirDelta2 in various species of rhinolophid bats. The results of PCR-based screening of selected Rhinolophus DNA samples are shown in a consensus species cladogram. Species that tested positive for ChirDelta2 sequence are in blue, species that tested negative are in red. The monophyletic group, comprising the positive species (marked by blue branches), defines the branch where integration of ChirDelta2 probably occurred. Time estimates for nodes surrounding this branch are shown. Geographical distributions of the species are indicated on the right side.

Assuming that the presence of the same ERV copy in related species is an evidence that integration has occurred in their common ancestor, the time calibrated phylogeny of positive and negative *Rhinolophus* species can provide an estimate of the time since ChirDelta2 integration age. We reconstructed a consensus species cladogram according to current knowledge of Rhinolophidae phylogenetic relationships [12,13,14,15,16]. In the cladogram taxa formed two separate groups, within which species were either all positive or all negative for the ChirDelta2 insertion (Figure 2). This is in agreement with the assumption that ChirDelta2 sequence in rhinolophids represents a single integration event. Based on the estimated diversification times for particular rhinolophid species presented in Dool et al. [12], the integration of ChirDelta2 occurred between 10.99 and 18.59 million years ago (MYA).

**Figure 3:**
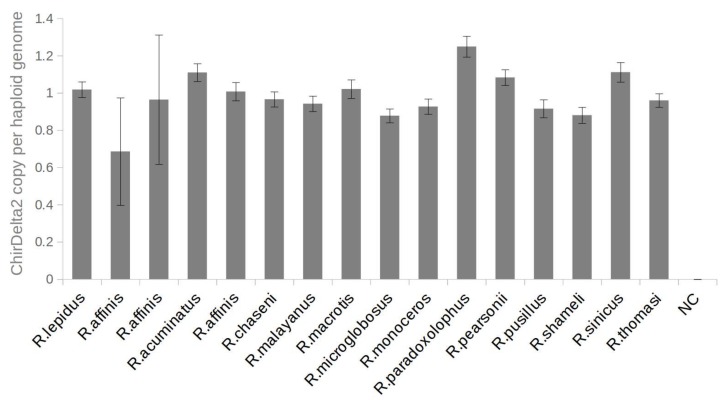
Number of ChirDelta2 copies in genomes of rhinolophid species. The chart shows copy numbers of ChirDelta2 sequence. These were determined by ddPCR absolute quantification using a set of primers specific to the internal part of the ChirDelta2 sequence. All values were normalized to the values obtained for reference locus in rhinolophid genome. The error bars represent poisson 95% confidence intervals of ddPCR analysis. NC—nontemplate control.

The findings described above indicate that only a single ChirDelta2 copy is present in rhinolophid species. To confirm this, we used digital droplet PCR (ddPCR): an emulsion PCR method enabling highly accurate DNA quantifications. The ddPCR analysis showed that all positive rhinolophid species possess two alleles of ChirDelta2 per diploid genome as expected (Figure 3). The small deviations from a theoretical diploid copy-number presumably reflect variation in sample quality.

## 4. Discussion

In this study we describe the second case of putative endogenous *Deltaretrovirus*, present in the genome of horseshoe bats. This provirus, ChirDelta2, is represented by a solo LTR, present in a single diploid copy in the host genome.

Retroviral LTR sequences are in general highly variable [8], e.g., even closely related deltaretroviruses such as HTLV1, 2, 3 and 4 exhibit only about 45–55% sequence identity in LTR region. In the case of ChirDelta2, we detected 53.6% identity to the LTR of MINERVa—the only previously described endogenous *Deltaretrovirus.* No other significant match to both retroviral sequences and vertebrate genomes was detected. This suggests that the ChirDelta2 integration is derived from an exogenous *Deltaretrovirus* related to MINERVa, which infected the ancient rhinolophids. Additionally, further lines of evidence support the deltaretroviral origin of ChirDelta2. These include the high cytosine content and the presence of the splice donor motif in LTR, both typical for deltaretroviruses.

The lack of internal sequences, including viral genes, strongly limits the scope of the analysis that can be performed here. For example, we cannot exclude the possibility that the original virus was not of deltaretroviral origin, and that its LTR sequence was generated by recombination with MINERVa-like virus.

Interestingly, the ChirDelta2 represents an orthologous integration present only in a subset of rhinolophid species. This means that integration occurred during rhinolophid diversification, in contrast to MINERVa, which integrated before the radiation of their host species, miniopterid bats [3]. The screening for the ChirDelta2 positive and negative species, therefore, makes it possible to more precisely determine where in the rhinolophid phylogeny the integration occurred. Here, we used a species cladogram reconstructed according to recent literature. Indeed, all positively tested species form a monophyletic group, separated from the all negative species (Figure 2). Assuming that ChirDelta2 integration occurred in a common ancestor of the positive species, this confirms the species relationships presented in the cladogram. Generally, large insertions or deletions, as reported in the present study, may help to reveal monophyletic groups within the framework of Camin-Sokal parsimony [17], as illustrated also in the mammalian phylogeny. For example, nine-base-pair deletion in the BRCA1 gene provides support for the monophyly of the clade Afrotheria [18] or ancient retroposon insertions supports the Laurasiatherian clade Pegasoferae [19].

The distribution of ChirDelta2-positive and negative rhinolophid species in the phylogeny and time estimates presented in the Dool et al. [12], indicate that ChirDelta2 was incorporated into the germline between 11 and 19 MYA. Interestingly, this interval is close to the time estimate of MINERVa infiltration into the Miniopteridae (20–45 MYA) [3]. However, because individual bat phylogeny estimates are not in complete agreement, the temporal relationships of these two ancient deltaretroviral lineages remain approximate. Putative endogenous deltaretroviruses have now been identified in two bat families with different evolutionary histories (being parts of two independent radiations of bats that occurred in early Paleocene [20]). This finding illustrates the potential of bats, which represent almost quarter of mammalian species, to serve as virus hosts and vectors [21,22]. Bat’s active heterothermy is connected with oscillation of body temperature, with lower immune response during torpor stage and potential selection of viruses to fever resistance by temperature increases during active flight. Relaying on interferons instead of CD8+ T killer cells is often considered to be a cause of a rather latent course of viral infections in bats [23]. Additionally, virus transfer might be facilitated by the bat longevity (generally in tens of years) and high dispersal capacity due to the ability of active flight. Moreover, frequent formation of mass polyspecific aggregations in tropical caves facilitates virus transfers and enables the viruses to survive in the cave aerosol much longer than in outer environments.

**Figure 4:**
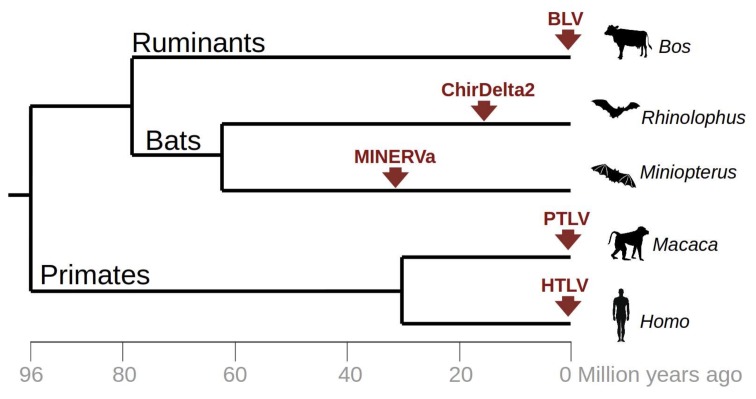
Occurrence of currently known retroviral infections related to *Deltaretrovirus* genus. The tree represents a chronogram of vertebrate species in which an evidence of deltaretrovirus infection has been documented. Red arrows indicate time of infection for particular viral lineages including both exogenous deltaretroviruses (BLV, HTLV, PTLV) and endogenous viral fragments putatively related to deltaretroviruses (MINERVa, ChirDelta2). The chronogram was reconstructed based on the time estimates from timetree.org.

The discovery of ChirDelta2 in rhinolophid bats extends the currently known set of endogenous (MINERVa) and exogenous (HTLV/PTLV, BLV) deltaretroviruses, which are harbored in hosts belonging to three mammalian orders (Figure 4). Both endogenous infiltrations have been described in bats, pointing to a close association of deltaretroviruses with bat ancestors. Currently, fourteen bat genome sequences representing seven families are publicly available. The rapid progress of the Bat 1 K genome initiative [24] promises to deliver the genomes of all approximately 1300 bat species, representing 21 families. This might reveal additional (and hopefully more complete) sequences of ancient deltaretroviruses. These findings also highlight the need to carefully screen the current metagenome data from bats for the presence of a circulating complex retrovirus and for more analysis of evolution of this enigmatic retroviral genus.

## Supplementary Materials

The following are available online at http://www.mdpi.com/1999-4915/10/4/185/s1, Figure S1: Annotation of ChirDelta2 sequence present in R. sinicus genome assembly, Figure S2: DNA sequence alignment of ChirDelta2 and MINERVa LTR, Figure S3: Comparison of cytosine content in retroviral LTR sequences, Table S1: List of the bat species analyzed.
