# Supplementary material for: Remnants of an Ancient Deltaretrovirus in the Genomes of Horseshoe Bats (Rhinolophidae)
Source: Viruses. 2018 Apr 10;10(4):185. doi: 10.3390/v10040185 (PMC5923479; doi:10.3390/v10040185)
Supplement: Supplementary file 1 [file Figure_S2.pdf › Figure_S2.pdf]

```
Reference sequence (1): MINERVa
Identities normalised by aligned length.
Colored by: identity + property

                      1 [        .         .         .         .         :         .         .         . 80
1 MINERVa     100.0%    TGTAGCTGAAGGGCCCTCCATGGAGG----GGCAAGA-----AGAAGGAAGTCAGAGCAGACAGCGGAAACCGAAGGAGC
2 ChirDelta2   53.6%    TGTAATGGAG-----CATCATGGGGGACTTGACTTGATTTCCCGAAGCTGTTCGTGACTGACCTTCTACACT-------C

                     81          .         1         .         .         .         .         :         . 160
1 MINERVa     100.0%    CATGAATAACTG--AGCCACGTACTTGTGGC-----------------------AGGATGAAGGCCAG------------
2 ChirDelta2   53.6%    CAAAACCACCTGACAGACACAGGCCTGCAGCCCACATACGAAAAATATCTGCGTGGGAAGATCATCAGCACCTTCAGCCG

                    161          .         .         .         2         .         .         .         . 240
1 MINERVa     100.0%    ------CCTGCATGGCTGGTGACCT-GTGACATCCTCTTAGCAACAACCCAG------ATATGTTTCCCCATAC-CTTGC
2 ChirDelta2   53.6%    TCATATCCTGCGCTGCCGACAAGATGGCCACAATTTCCCAGGACTAACCCCGCCAGACAAATACTTCCCATTGCTCAGGC

                    241          :         .         .         .         .         3         .         . 320
1 MINERVa     100.0%    CATCAC-------------GCTATGGCATTTAGCATACCCC------GAGATACCCAAATTACCTTCCTGTTAACCTCAA
2 ChirDelta2   53.6%    TACCATGAATCCTTTTGAGGTTGTGACAT---AGATACCCCTGCTTTGCAATTTCCCCATTCCTGTGCGGTTTTACCTAA

                    321          .         .         :         .         .         .         .         4 400
1 MINERVa     100.0%    TAAAATGCCGGTCCACG--GGTGGCAGGGGACTCAGAAGCGA-----CGCTAGGCACCGAAGGCCTAGCTCCCCGTTCGG
2 ChirDelta2   53.6%    TAAAATGCCAGTCCACTTCGGCGACAGGG--CTCAGAAGCGAATGTCCTGTGTATAACGCAGGCAT---TCCCCGTCCGG

                    401          .         .         .         .         :         .         .         . 480
1 MINERVa     100.0%    GTTCTGGTACTCTCGAGACGCTCGTGGCCACGGG-ACTAGATCTCCCAGAACTTCTGAGTCGCCGCTGGCCAGGTAAGCT
2 ChirDelta2   53.6%    GTTCTGGTCCTCTTGAGATGCTCGAGGCCTCAAGTCCTAAGTCTCCCAGAACTTCTGAGT-GCTGCCCACCAGGTTGACT

                    481          .         5         .         .         .         .         :         . 560
1 MINERVa     100.0%    CCCCGCCGATTGACAGGCCTCCTCCGGCCCGGCCTGGCCAGGCCGTTGAGTTGGTTGCTCTCTTGCCTAGTCGCTCGACT
2 ChirDelta2   53.6%    TCCC-CCGGTTGACACTCTACCACCGGCCTGACCAGGCCAGTCGGTTGAG------GCCTCTGTCCCAAACCGCTAGACT

                    561          .         .         .         6         .         .         .         . 640
1 MINERVa     100.0%    -AGTGTCTCGG---TGTCTTGTTGGGACTTGCCTGCC--CCTGTCGCTCACGGACGCCGGGCTTAAAATTCTCACTTCCA
2 ChirDelta2   53.6%    CGGTGTCCTGGGCTTCTCTTTCCAGAAGTTCTCTGCCTGCCTGTCGCTGACGGATGTCTAGCTTAAA---CTCACT----

                    641          :         .         .         .         .         7] 701
1 MINERVa     100.0%    CGTCTG-----TGTCTTTTACTTTCAGTTCGCCGTGTAACAGAGTCCGGGACTTTGTTACA
2 ChirDelta2   53.6%    CGTTTAGCTTCCGCCTCTTACTTTCGGTTTAGATTGTTGGCCTCCCCAGGACACTA--ACA
```

Figure S2: DNA sequence alignment of ChirDelta2 and MINERVa LTR.
DNA sequence alignment was generated using Muscle algorithm. Calculation of percent identity and visualization was performed by MView tool (www.ebi.ac.uk). MINERVa - consensual sequence of MINERVa orthologs [3]; ChirDelta2 - sequence from *R. sinicus* genome assembly (GenBank accession no. LVEH00000000).
