# Supplementary material for: Remnants of an Ancient Deltaretrovirus in the Genomes of Horseshoe Bats (Rhinolophidae)
Source: Viruses. 2018 Apr 10;10(4):185. doi: 10.3390/v10040185 (PMC5923479; doi:10.3390/v10040185)
Supplement: Supplementary file 1 [file viruses-10-00185-s001.zip › FigureS1.pdf]

Rhinolophus sinicus genomic scaffold LVEH01002092.1:218-893
(Accession number:start-end)

target site duplication

proviral LTR sequence

TTTATC**TG**TAATGGAGCATCATGGGGGACTTGACTTGATTTCCCGAAGCTGTTCGTGACTGACCTTCTACACT

CCAAAACCACCTGACAGACACAGGCCTGCAGCCCACATACGAAAAATATCTGCGTGGGAAGATCATCAGCACC

TTCAGCCGTCATATCCTGCGCTGCCGACAAGATGGCCACAATTTCCCAGGACTAACCCCGCCAGACAAATACT

TCCCATTGCTCAGGCTACCATGAATCCTTTTGAGGTTGTGACATAGATACCCCTGCTTTGCAATTTCCCCATT

polyadenylation signal site

CCTGTGCGGTTTTACCTAATAAAATGCCAGTCCACTTCGGCGACAGGGCTCAGAAGCGAATGTCCTGTGTATA

ACGCAGGCATTCCCCGTCCGGGTTCTGGTCCTCTTGAGATGCTCGAGGCCTCAAGTCCTAAGTCTCCCAGAAC

splice donor site

TTCTGAGTGCTGCCCACCAGGTTGACTTCCCCCGGTTGACACTCTACCACCGGCCTGACCAGGCCAGTCGGTT

GAGGCCTCTGTCCCAAACCGCTAGACTCGGTGTCCTGGGCTTCTCTTTCCAGAAGTTCTCTGCCTGCCTGTCG

CTGACGGATGTCTAGCTTAAACTCACTCGTTTAGCTTCCGCCTCTTACTTTCGGTTTAGATTGTTGGCCTCCC

target site duplication

CAGGACACTAA**CA**TTTATC

Figure S1: Annotation of ChirDelta2 sequence present in R. sinicus genome assembly. The donor splice site was predicted using the NNSPLICE 0.9 algorithm (www.fruitfly.org/seq_tools/splice.html).
