# Supplementary material for: Remnants of an Ancient Deltaretrovirus in the Genomes of Horseshoe Bats (Rhinolophidae)
Source: Viruses. 2018 Apr 10;10(4):185. doi: 10.3390/v10040185 (PMC5923479; doi:10.3390/v10040185)
Supplement: Supplementary file 1 [file viruses-10-00185-s001.zip › Figure_S3.pdf]

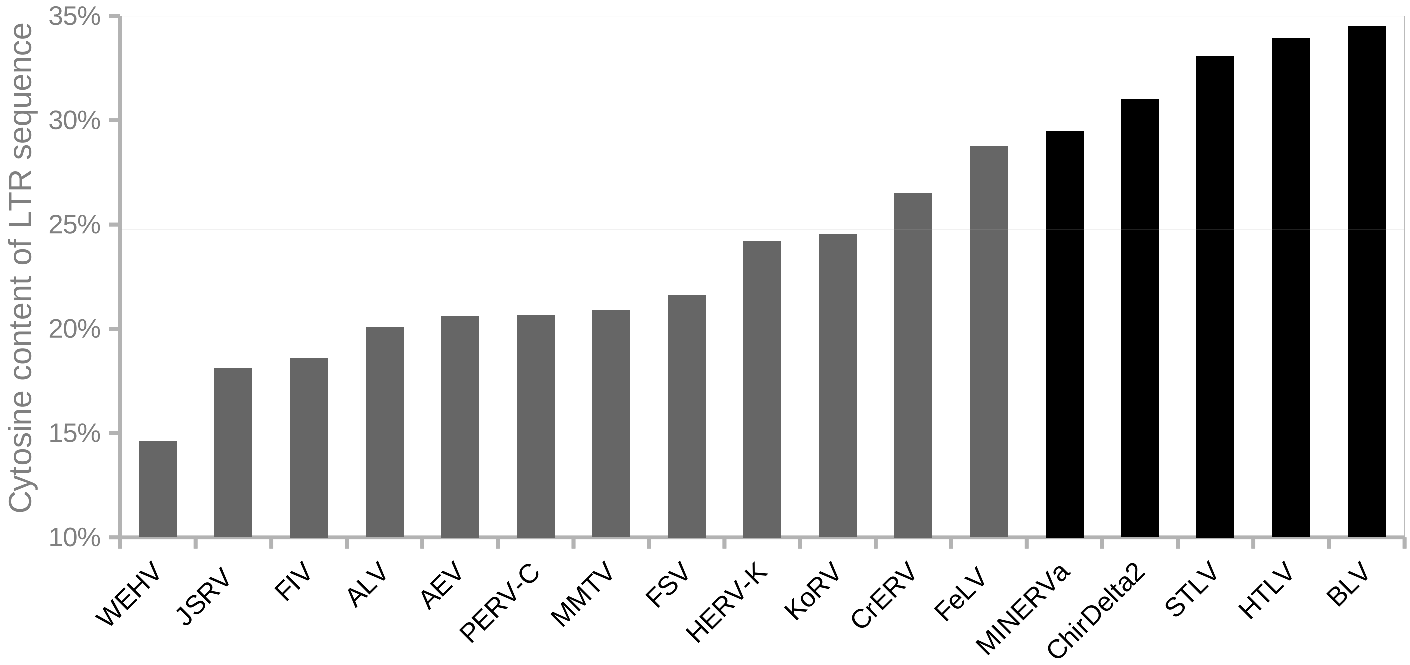

Figure S3: Comparison of cytosine content in retroviral LTR sequences.
Cytosine content in LTR sequences of various retroviruses is shown. Black columns represent viral sequences belonging to genus Deltaretrovirus. Horizontal line represents average cytosine content for all sequences shown in the chart. Alpharetrovirus: ALV (AC:AY013305), AEV (AC:K03326), FSV (AC:J02194); Betaretrovirus: MMTV (AC:X98457), HERV-K (AC:Y17834), JSRV (AC:A27950); Gammaretrovirus: PERV-C (AC:AJ298075), KoRV (AC:KJ152815), CrERV (AC:KP261824), FeLV (AC:M12500); Deltaretrovirus: MINERVa (AC:KY250078), STLV (AC:M33063), HTLV (AC:AH002564), BLV (AC:K02120); Lentivirus: FIV (AC:M25381); Epsilonretrovirus: WEHV (AC:AF133052).
