# Supplementary material for: Remnants of an Ancient Deltaretrovirus in the Genomes of Horseshoe Bats (Rhinolophidae)
Source: Viruses. 2018 Apr 10;10(4):185. doi: 10.3390/v10040185 (PMC5923479; doi:10.3390/v10040185)
Supplement: Supplementary file 1 [file viruses-10-00185-s001.zip › TableS1.pdf]

Scientific name	Common name	Family	original	origin
Rhinolophus blasii	Blasius' horseshoe bat	*Rhinolophidae*	NMP 90930	Cyprus, southern part, Troodos Forest
Rhinolophus ferrumequinum	greater horseshoe bat	*Rhinolophidae*	NMP 91235	Cyprus, northern part, Afendrika
Rhinolophus hipposideros	lesser horseshoe bat	*Rhinolophidae*	NMP 91263	Cyprus, southern part, Troodos Forest
Rhinolophus lepidus	Blyth's horseshoe bat	*Rhinolophidae*	NMP 95762	Tajikistan, Dushanbe Prov., Magov
Rhinolophus affinis	intermediate horseshoe bat	*Rhinolophidae*	NMP 90558	China, Shaanxi Prov., Daguping
Rhinolophus affinis	intermediate horseshoe bat	*Rhinolophidae*	NMP 90564	China, Shaanxi Prov., Daguping
Rhinolophus affinis	intermediate horseshoe bat	*Rhinolophidae*	HNHM 24996	Vietnam, Thanh Hoa Prov., Ban Vin Village, Xuan Lien NR
Rhinolophus acuminatus	acuminate horseshoe bat	*Rhinolophidae*	HNHM 23675	Cambodia, Preah Vihear Prov., Preah Vihear Protected Forest
Rhinolophus chaseni	Noc Dao horseshoe bat	*Rhinolophidae*	HNHM 2011.15.1.	Cambodia, Rattanakiri Prov., Veun Sai District, Veun Sai Forest
Rhinolophus formosae	Formosan wooly horseshoe bat	*Rhinolophidae*	HNHM 25783	Taiwan, Hualien, Taroko NP
Rhinolophus luctus	wooly horseshoe bat	*Rhinolophidae*	HNHM 25323	China, Jiangxi Prov., Jiulianshan Reserve
Rhinolophus macrotis	big-eared horseshoe bat	*Rhinolophidae*	HNHM 25892	China, Yunnan Prov., Dali, Qinghua Gudong Cave
Rhinolophus malayanus	Malayan horseshoe bat	*Rhinolophidae*	HNHM 2007.50.29.	Vietnam, Quang Tri Prov., Da Krong Nature Reserve
Rhinolophus microglobosus	Indo-Chinese lesser horseshoe bat	*Rhinolophidae*	HNHM 2007.49.13.	Cambodia, Pusat Prov., Pramaoy, Phnom Samkos
Rhinolophus monoceros	Formosan lesser horseshoe bat	*Rhinolophidae*	HNHM 2016.19.4.	Taiwan, Hualien, Taroko NP
Rhinolophus paradoxolophus	Bourret's horseshoe bat	*Rhinolophidae*	HNHM 26000	Vietnam, Hoa Binh Prov., Xom Khu, Ngoc Son Ngoc Luong NR
Rhinolophus pearsonii	Pearson's horseshoe bat	*Rhinolophidae*	HNHM 25970	Vietnam, Hoa Binh Prov., Xom Khu, Ngoc Son Ngoc Luong NR
Rhinolophus pusillus	least horseshoe bat	*Rhinolophidae*	HNHM 25002	Vietnam, Thanh Hoa Prov., Ban Vin Village, Xuan Lien NR
Rhinolophus shameli	Shamel's horseshoe bat	*Rhinolophidae*	HNHM 23674	Cambodia, Preah Vihear Prov., Preah Vihear Protected Forest
Rhinolophus sinicus	Chinese horseshoe bat	*Rhinolophidae*	HNHM 25977	Vietnam, Hoa Binh Prov., Xom Khu, Ngoc Son Ngoc Luong NR
Rhinolophus thomasi	Thomas' horseshoe bat	*Rhinolophidae*	HNHM 25013	Vietnam, Thanh Hoa Prov., Ban Vin Village, Xuan Lien NR
Rhinopoma hardwickii	Indian mouse-tailed bat	*Rhinopomatidae*	NMP 48163	Iran, Khuzestan Prov., Izeh, Sarkan
Rhinopoma muscatellum	Muscat mouse-tailed bat	*Rhinopomatidae*	NMP 48171	Iran, Khuzestan Prov., Izeh, Sarkan
